# Ventilator-associated pneumonia in critically ill patients with intensive antibiotic usage

**DOI:** 10.12669/pjms.316.8038

**Published:** 2015

**Authors:** Canan Bor, Kubilay Demirag, Ozlem Okcu, Ilkin Cankayali, Mehmet Uyar

**Affiliations:** 1 Canan Bor, Department of Anaesthesiology and Intensive Care Unit, Ege University School of Medicine Hospital, Izmir, Turkey; 2 Kubilay Demirag, Department of Anaesthesiology and Intensive Care Unit, Ege University School of Medicine Hospital, Izmir, Turkey; 3 Ozlem Okcu, Department of Radiology, Ege University School of Medicine Hospital, Izmir, Turkey; 4 Ilkin Cankayali, Department of Anaesthesiology and Intensive Care Unit, Ege University School of Medicine Hospital, Izmir, Turkey; 5 Mehmet Uyar, Department of Anaesthesiology and Intensive Care Unit, Ege University School of Medicine Hospital, Izmir, Turkey

**Keywords:** Clinical Pulmonary Infection Scores (CPIS), Mortality, Ventilator-associated pneumonia (VAP)

## Abstract

**Objective:**

Ventilator-associated pneumonia (VAP) is an infection with high mortality and morbidity that prolongs the length of stay in the intensive care unit (ICU) and hospitalisation. VAP is one of the most common infections in critically ill patients. This study aimed to prospectively determine the VAP rate and associated factors in critically ill patients with intensive antibiotic usage during a one-year period.

**Methods:**

In total, 125 out of 360 patients admitted to the intensive care unit during the one-year study period (September 2010-2011) were included for follow-up for VAP diagnosis. Demographic data, APACHE II scores, diagnoses on admission, clinical pulmonary infection scores (CPIS), CRP, procalcitonin, risk factors for infection, time to VAP diagnosis, and bacteriological culture results were recorded. All data were assessed in terms of ICU, hospital and 28-day mortality.

**Results:**

In total, 56 (45%) out of 125 patients were diagnosed with VAP. In addition, 91% of patients diagnosed with VAP were administered antibiotics before diagnosis. In the VAP patients, the mortality rates were 48, 68 and 71% for 28-day, ICU and hospital mortality, respectively.

**Conclusion:**

The coexistence of clinical and microbiological parameters should not be sought when diagnosing VAP in patients who use antibiotics intensively. VAP can be diagnosed when CPIS≤6 in cases with sufficient microbiological evidence. This strategy may decrease mortality by preventing a delay in therapy.

## INTRODUCTION

Ventilator-associated pneumonia (VAP) is defined as a pulmonary infection developing after 48 hours following intubation. The condition leads to a prolonged duration of ventilatory support and increased morbidity and mortality rates.^1^,^2^

Significant differences in VAP rates have been reported in the literature.^3^-^6^ The reasons for this variability include a lack of standard diagnostic criteria, methodological differences, variations in study populations, differences in the evaluation of radiological images of pneumonia (especially in trauma patients with pulmonary contusion and acute lung injury) and different methods of microbiological sampling.^7^

Despite the fact that microbiological support was demonstrated to be useful once VAP was clinically and radiologically diagnosed, no agents were detected in almost half of the cases.^8^ Based on autopsy results, 29-62% of the patients were misdiagnosed.^9^ Although findings, such as fever, leucocytosis, purulent sputum, localised infiltrations based on chest radiography, exhibit high sensitivity, they have low specificity for the diagnosis of VAP.^10^ Microbiological sample results are obtained between 24 and 72 hours; however, these results could be falsely negative due to antibiotics used by the patient.^11^ Clinical pulmonary infection scores (CPIS), which use clinical and laboratory diagnosis criteria for VAP diagnosis, were created by Pugin et al. in early 1990s, and the scoring system is widely used.^12^-^14^

CPIS assesses body temperature, degree of leukocytosis, nature of tracheal secretions, arterial oxygenation (PaO_2_:FiO_2_ ratio), evidence of infiltration on chest radiography, changes in chest radiographs and results of tracheal aspirate cultures as diagnostic variables in determining the likelihood of VAP. A point system is used to determine this likelihood, and a score of greater than six points is considered suspicious for the diagnosis of VAP.

Given their primary pathologies, patients admitted to our unit present with increased antibiotic use. In this study, we aimed to prospectively analyse VAP incidence, VAP risk factors, intensive care and hospital mortality. Our secondary aim was to assess the correlation among CRP and procalcitonin levels and VAP diagnosis.

## METHODS

After approval by our institutional ethics committee, all patients over 18 years old who were intubated and mechanically ventilated were included. Patients having immunosuppressive therapy, neutropenia, malignancy on chemotherapy, lung cancer, pneumonia, pulmonary oedema, pulmonary fibrosis, or AIDS were excluded. Patients previously intubated or tracheotomised in other clinics and readmitted patients were excluded.

During this one year study (September 2010- 2011), daily recordings of body temperature, respiratory secretion characteristics and PaO_2_/FiO_2_ ratios were noted. Leukocyte counts were assessed thrice weekly, and chest radiography was performed twice weekly. Mini-BAL was assessed when the patient’s body temperature was greater than 38.3ºC twice consecutively during follow-up. Mini-BAL was sent to the laboratory within 30 minutes after the patient was administered 20 ml of saline and at least 2 ml was aspirated. Bacterial growth was considered to be positive when significant bacterial populations (≥ 10^4^ CFU/ml) were detected by routine laboratory evaluation. Using these values, CPIS scores for all study patients were calculated daily by an intensivist. Chest radiographies were assessed by a radiologist.

In addition to CPIS score parameters, CRP and procalcitonin levels, gender, age, diagnosis on admission, duration of intensive care, BMI (body mass index), Glasgow Coma Scale before intubation, APACHE II score, administered antibiotics and duration of administration, mini-BAL results, other possible infections (blood, urine, wound, abdominal drain, or catheter), nutritional route (enteral-parenteral or combination), previous abdominal and thoracic operations, head trauma and mortality were noted. Extubated patients were monitored for VAP for 48 hours following extubation. When the CPIS score was greater than 6 and/or a positive mini-BAL culture (≥ 10^4^ CFU/ml) was noted, the patient was diagnosed with VAP. Only the first VAP attack was considered; repeated attacks were not considered. Follow-up was stopped after VAP diagnosis or if the patient did not develop pneumonia within 28 days. Extubated patients were monitored for VAP for 48 hours following extubation. Antibiotic therapies initiated in previous clinics were continued or altered if necessary during the follow-up.

Patient outcomes were also followed at other clinics where patients were transferred until hospital discharge and all complications were noted.

### Statistical Analysis

All statistical analyses were made using SPSS 20.0 by Ege University Medical School Department of Information and Statistics. The Shapiro-Wilk test was used if the numerical data fit a normal distribution. For comparison of different groups, Mann-Whitney U-tests or Student’s t-tests were used. When comparing categorical data, Chi-Square or Fisher’s exact tests were used. ROC analyses were performed to obtain cut-off values. All data were expressed as absolute values, percentages, median (± IR) or means (± standard deviation) as appropriate. A 2-tailed probability value of ≤ 0.05 was accepted as statistically significant.

## RESULTS

**Fig.1:**
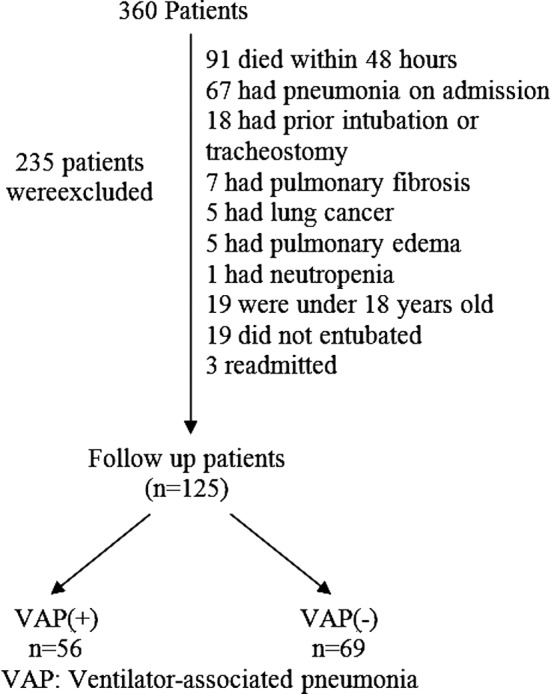
Flow chart representing the study design.

In total, 360 patients were admitted to the intensive care unit within a one-year period, of which 235 patients were excluded from the study (Fig.1). Patient characteristics and VAP risks factors are provided in Tables I and II. Intensive care unit and hospital mortality of the patients with an APACHE score greater than 21 were significantly increased compared with patients with a score lower than 21 (P=0.0017).

**Table-I:** Characteristics of the patients at enrollment (n=125).

	VAP(+) (n=56) mean	VAP(-) (n=69) mean	P
Age (years)	61±18	51±20	0.003*
BMI	27±6	27± 5	0.646
APACHE II	21.1±7.5	22.0±8.7	0.724
CPIS	6.58±1.1	3.17±1.7	0.0001

* p<0.05, BMI: Body Mass Index.

APACHE: Acute Physiology and Chronic Health Evaluation. CPIS: Clinical Pulmonary Infection Scores, VAP: Ventilator-associated pneumonia.

**Table-II:** Characteristics of the study groups at enrollment.

	VAP(+) (n=56) n (%)	VAP(-) (n=69) n (%)	p
Sex(M)	24(43)	30(43)	1.0
Medical	13(52)	12(48)	0.496
Surgical	30(46.2)	35(53.8)	0.834
Trauma	13(37.1)	22(62.9)	0.494
Abdominal surgery	22(53.7)	19(46.3)	0.184
Enteral nutrition	23(41)	40(58)	0.158
Parenteral nutrition	26(46)	24(35)	0.158
Combined enteral +parenteral	7(12.5)	5(7)	0.158
Other infection	13(23.2)	5(7.2)	0.033*
Antibiotic usage	51(91.1)	58(84.1)	0.286
28-day mortality	27(48.2)	29(42)	0.588
ICU mortality	38(67.9)	35(50.7)	0.068
Hospital mortality	40(71.4)	38(55.1)	0.066

VAP: Ventilator-associated pneumonia.

**Fig.2:**
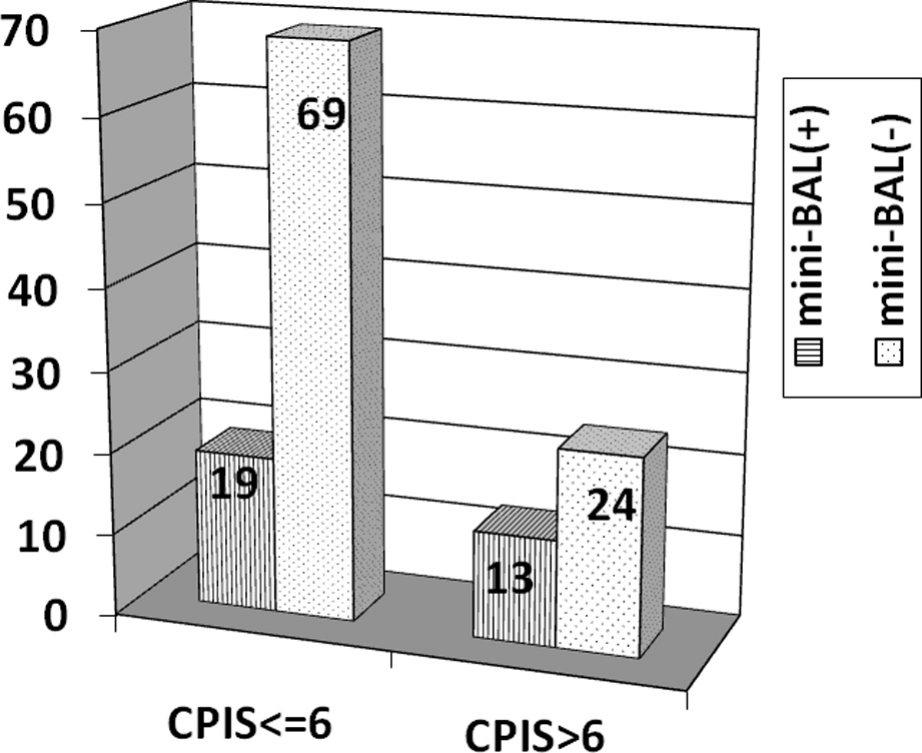
CPIS and microbiological identification in mini-BAL.

In total, 56 (45%) of the cases were diagnosed with VAP. Six of the diagnosed patients (10.7%) had early VAP, and 50 of the patients (89.2%) had late VAP (≥ 4 days). The time to VAP diagnosis was 9 ± 4 days. In all cases, the antibiotic use rate was 87.2% on admission and 91.1% in patients with VAP. In VAP patients, the most common infection outside the lung was bacteraemia (23.2%) followed by abdominal infection (8.9%). Only 32 (57.1%) of the VAP patients had positive mini-BAL culture. Responsible microorganisms in VAP (+) patients are provided in Table-III. All early VAP cases had positive mini-BAL cultures, whereas 48% of late VAP patients had positive cultures (P=0.003). *Methicillin-sensitive Staphylococcus aureus (MSSA)* was the major bacterium grown in early VAP cases, whereas *Methicillin-resistant Staphylococcus aureus (MRSA)* and *Acinetobacter baumannii* were the most common bacteria in late VAP. No significant differences in VAP (+) and (-) patients were noted in terms of diagnosis on admission, BMI, nutritional status, previous abdominal operations, CRP and procalcitonin levels. The relationship between microbiological growth and CPIS is presented in Fig.2. Despite having CPIS scores ≤ 6, 19 patients were diagnosed with VAP due to positive mini-BAL cultures. The rate of VAP development was significantly increased in elderly patients (P=0.003). In patients who developed VAP, the 28-day mortality rate was 48.2%, the ICU mortality rate was 67.9% and the hospital mortality rate was 71.4%. Although mortality rates were increased in VAP (+) patients, the difference was not statistically significant (P>0.05).

**Table-III:** Responsible microorganisms in Ventilator-associated pneumonia (+) patients.

Responsible microorganism	(n=32)
Acinetobacter baumannii	17
MRSA (Methicillin resistant S.aureus)	6
Pseudomonas aeruginosa	4
MSSA (Methicillin sensitive S.aureus)	4
Haemophilus influenzae	1

## DISCUSSION

Pneumonia is common in mechanically ventilated patients. The condition requires detailed assessment of various factors for diagnosis and exhibits high morbidity and mortality rates. No gold standard is available for diagnosis even though some criteria have been developed to increase diagnostic accuracy. Although several criteria, scoring systems and sampling methods are available, final diagnostic criteria have not been successfully developed, especially for critically ill patients with multiple pathologies. Based on autopsy results, only 43-75% of VAP (+) patients were confirmed histologically, whereas 31-46% of VAP diagnoses were missed.^15^ The CPIS scoring system developed by Pugin increases the specificity of the clinical diagnosis of pneumonia, and a bacterial correlation with CPIS > 6 has been noted.^16^ Therefore, we used this scoring system in the current study. Cases supported microbiologically and/or clinically through CPIS were considered to have VAP. In total, 21% (19/88) of the patients with CPIS ≤ 6 and 35% (13/37) of patients with CPIS > 6 had positive mini-BAL cultures (P=0.08). Some difficulties are encountered when diagnosing pneumonia using the CPIS scoring system in mixed ICUs. Previous antibiotic usage, surgical interventions necessitating antibiotics, non-pulmonary infections causing fever and leukocytosis, increased secretion due to local reaction to the endotracheal tube, immunocompromised patients, low quality chest radiographies, concurrent atelectasis and pleural pathologies are among the difficulties encountered when diagnosing VAP.^17^ Some researchers compared CPIS results with post-mortem findings and culture-antibiogram results and reported 77% sensitivity and 42% specificity.^16^ CPIS is more reliable in medical and surgical patients compared with trauma and burn patients.^18^,^19^ Due to the above reasons, the CPIS scoring system may be considered unsuitable for mixed ICU populations, such as those in our study. Mean age (61 ± 18) was significantly increased in VAP (+) patients. Advanced age is an important factor regarding mortality and development of many diseases given that age depresses the immune system.^20^ These patients do not react to fever, thus delaying the diagnosis.^21^,^22^

Empirical antibiotic use was reported as a confounding factor for infection diagnosis. The percentage of empirical antibiotic use was 34-74.4% in the general ICU population.^4^,^23^ Bacteriological samples might provide false negative results when obtained while the patients are receiving antibiotics. Garrard et al. reported that these results are related to previous antibiotic use but not to the method used; they emphasised that colony count could be below the threshold value.^24^ Given that antibiotic usage was as high as 91% in our VAP (+) population, microbiologically supported VAP diagnoses were low despite regular mini-BAL sampling in all patients.

In other studies, 10-40% false negative result were reported when antibiotics were used; late VAP with resistant bacteria was more common.^7^,^21^ Under these circumstances, bacterial growth is not obligatory for pneumonia diagnosis, and this finding indicates the importance of clinical diagnosis and close monitoring.

The patient group with high APACHE II scores exhibited increased use of multiple antibiotics during the admission.^25^,^26^ In parallel, the APACHE II scores in our patients with intensive antibiotic use during admission were also increased.

The mortality of VAP patients ranges between 24-76%. The causes of this wide range include heterogeneous patient populations, insufficient study designs and diagnostic criteria and laboratory mishaps.^10^,^11^ There are conflicting results regarding mortality. Some studies report higher mortality rates in VAP (+) patients compared with controls, whereas others indicate no significant differences, similar to our study.^27^,^28^

An APACHE II score > 21 was significantly related to mortality in our study (P=0.0017). In previous studies, APACHE II scores were demonstrated to be useful for predicting mortality in VAP (+) patients, and an APACHE II score greater than 27 was shown to be an independent factor for mortality.^29^

In addition to clinical and microbiological data, some studies hypothesise that CRP and procalcitonin can be used to diagnose VAP.^30^-^32^ However, additional contradictory studies have also been reported.^33^ Accordingly, no statistically significant differences were identified between VAP (+) and VAP (-) patients in terms of CRP and procalcitonin values in our study.

We conclude that when diagnosing VAP in a patient with intensive antibiotic use, the coexistence of clinical and microbiological parameters are not compulsory. If there is sufficient microbiological evidence, VAP can be diagnosed when CPIS ≤ 6. Waiting for this coexistence may delay diagnosis and therapy.

### Limitations of the study

It is necessary to assess a larger patient group with no previous antibiotic use. The mixed ICU population with a wide range of different pathologies might inhibit the assessment of exact VAP incidence rates.
